# Association between Rheumatic Disease Comorbidity Index and factors of poor prognosis in a cohort of 280 patients with rheumatoid arthritis

**DOI:** 10.1186/s41927-022-00308-5

**Published:** 2022-12-21

**Authors:** Aicha Ben Tekaya, Emna Hannech, Olfa Saidane, Leila Rouached, Selma Bouden, Rawdha Tekaya, Ines Mahmoud, Leila Abdelmoula

**Affiliations:** 1 Rheumatology Department, Charles Nicolle Hospital, 1007 Tunis, Tunisia; 2 Faculty of medicine of Tunis, University Tunis El Manar, Tunis, Tunisia

**Keywords:** Rheumatoid arthritis, Comorbidity, Index, Prognosis

## Abstract

**Background:**

Rheumatoid arthritis (RA) is commonly associated with higher rates of comorbidities. Recent recommendations highlight screening comorbidities during the disease course because of their impact on patients’ ability to function, on disease outcome, but also on treatment choices. Hence the interest of our study that aimed to quantify the impact of comorbidities among RA patients using a validated tool the Rheumatic Disease Comorbidity Index (RDCI) and to explore the association between comorbidities and disease characteristics.

**Methods:**

We conducted a cross-sectional study over 12 months period, including patients followed for an established RA according to the ACR/EULAR 2010 criteria and hospitalized in our rheumatology department. Patients’ characteristics and disease features were collected for each patient. Comorbidities were quantified using the RDCI. We looked for the association between RDCI and patients characteristics and RA parameters. Univariable and multivariable analysis were made.

**Results:**

They were 280 patients: 233 female (83.2%) and 47 male (16.8%) with a mean age of 58.07 (SD 11.12) years. The mean follow-up period was 14.74 (SD 1.63) years. Comorbidities were noted in 133 patients (47.5%). The mean comorbidity score measured by the RDCI was 1.05 (SD 1.23). RDCI was positively correlated with age (p < 0.001, r = 0.359). RA patients whose age of disease onset exceeds 40 years have significantly higher RDCI (1.8 (SD 1.3) [CI 95%: 1.36–1.88] vs. 1.5 (SD 1.2), p = 0.007). Moreover, RDCI was significantly associated with the presence pulmonary involvement (p < 0.001) and ocular involvement (p = 0.002). RDCI was also associated with erosive RA (p = 0.006), the presence of atlanto-axial dislocation (p = 0.014), and coxitis (p = 0.029). Regarding therapy regimen, RDCI was statistically increased in patients receiving bDMARDs compared to patients under csDMARDs (2.8 (SD 1.6) vs. 1.0 (SD 1.0), p = 0.021).

**Conclusion:**

In this study, comorbidity index was associated with signs of poor prognosis such as erosions, coxitis, and atlanto-axial dislocation. This confirmed the hypothesis that comorbidity can be a threat to the improvement in the long-term prognosis in RA patients.

## Highlights

Patients with rheumatoid arthritis have higher rate of comorbidity which may impair their quality of life and affects disease outcomes.

Rheumatic Disease Comorbidity Index (RDCI) is an excellent tool to predict extra-articular manifestations and signs of poor prognosis such as erosions, coxitis, and atlanto-axial dislocation.

An early screening and an adequate management of comorbidities may result in better disease outcomes.

## Background

Rheumatoid arthritis (RA) is a chronic inflammatory and autoimmune disease that affects about 1% of the population worldwide [1]. It is characterized by progressive joint erosions and destruction and it is responsible of extra-articular manifestations estimated to be around 20–40% [2]. The evidence supporting on an over representative of comorbidities in this population is in perpetual progress [3, 4]. Comorbidities are defined as “the presence of co-existing or additional diseases with reference to an initial diagnosis or with reference to the index condition that is the subject of study” [5]. They constitute a major concern due to their impact on patients’ ability to function, on the disease outcome, but also on the treatment choices in RA. Moreover, recent data suggest that RA patients with high comorbidities may be receiving suboptimal treatment [6]. Nowadays, the management of RA implicates the management of comorbidities [7, 8]. Thus, the screening of comorbidities in RA is crucial.

Few authors were interested in studying the impact of comorbidities on the RA outcomes. Comorbidities influence differently RA outcomes such as mortality, hospitalization, and disability [9]. In fact, cardiovascular and respiratory diseases exposed RA patients to frequent hospitalizations and may be life threatening [10]. However, depression is commonly associated with disability [11].

A number of comorbidity instruments have been created to assess comorbidities in patients with rheumatic disease [12]. Comorbidity indices are tools used to quantify the total burden of comorbidity and help in the identification of patients with worse prognosis in terms of heightened mortality, hospitalization risk as well as decline in health-related quality of life. There are no guidelines stratifying the use of those different tools in clinical practice. Many tools have been created such as the Charlson Comorbidity Index (CCI) and the Elixhauser Comorbidity Index (ECI) [12]. But, there are not specific to rheumatic diseases. The rheumatic disease comorbidity index (RDCI) was developed for use specifically in rheumatology patients and was considered as RA-specific indices [13]. It has been used in only few studies [13–18]. It showed his superiority to predict physical disability and mortality for RA patients [12]. However, the link between RDCI and different disease characteristics in particular structural damages and the occurrence of extra-articular manifestations as pulmonary involvement is not well known. This study aimed to quantify the impact of comorbidities among RA patients using a validated tool in RA (the RDCI) and to explore the association between comorbidities and disease characteristics.

## Methods

### Study design and patients

We conducted a cross sectional study including patients followed for an established RA according to the American College of Rheumatology/European League Against Rheumatism (ACR/EULAR) 2010 criteria [19] and hospitalized in the rheumatology department of the Charles Nicolle Hospital of Tunis, during the period 2020–2021. Patients with incomplete medical records were excluded from the study. Our locally appointed ethics committee “Charles Nicolle Hospital local committee” has approved the research protocol. This study complies with the Declaration of Helsinki.

## Data collection and investigated variables

Demographic features (sex, age) were recorded. The following disease characteristics were collected for each patient: disease duration, age at onset of RA, immunological profile for rheumatoid factor (RF) and for anti-citrullinated protein antibodies (ACPA), disease activity measured by the disease activity score 28 (DAS28) [20], functional impairment measured by the Health Assessment Questionnaire (HAQ), and extra-articular manifestations. Therapy regimens as for conventional synthetic Disease-modifying antirheumatic drugs (csDMARDs) and biologic DMARDs (bDMARDs) were also investigated. Radiographs of the hands, forefoot, pelvic, and of the cervical spine were checked the time of enrolment.

Comorbidities were quantified using the RDCI, which is a validated method to quantify comorbidities in RA [13]. It comprises 11 comorbid conditions including lung disease, cardio-vascular disease, hypertension, diabetes, fracture, depression, cancer, and gastrointestinal ulcer. Each comorbidity type was a binary variable, assigned a score of 0 or 1. However, lung disease, heart attack, stroke, and others cardiovascular conditions are given greater weight in the formula. The total RDCI score rated from 0 to 9. Patients having two or more of chronic comorbid conditions were classified in multimorbidity.

### Statistical analysis

We used SPSS software version 23.0 for statistical analysis. The categorical data were expressed as numbers and percentages and the continuous data as means and standard deviations. Data were normally distributed using the test Kolmogorov-Smirnov. We looked for the association between RDCI and patients characteristics and RA parameters. The Pearson’s χ2 test was used for studying the association between two independent qualitative variables. The correlation between two continuous variables was performed using the Pearson test and between two categorical variables using the Chi-squared test. The Student’s t-test was used to study the association between a categorical and a continuous variable. Welch’s Test was used for unequal variances. It was only applicable for atlanto-axial dislocation. Missing variables were addressed using imputation.

A multivariable analysis by means of multiple linear regression were performed to investigate which variables were independently associated with RDCI. This analysis included variables that were considered significant with a p-value of 0.2 or less in univariable analysis. The p-value was considered statistically significant when the value was less than 0.05.

## Results

### Clinical outcomes of RA patients

The RA cohort consisted of 280 patients. They were 233 female (F) (83.2%) and 47 male (M) (16.8%) with a sex ratio M/F at 0.2. Table 1 shows the main patients characteristics and disease features. The mean age was 58.07 (SD 11.12) years with extremes ranging from 25 to 85 years. They were 73 patients (26.1%) aged 65 years or over. The mean age at diagnosis of RA was 44.65 (SD 13.78) years. They were 53 patients (18.9%) with an age of RA onset over 40 years. The mean disease duration was 14.74 (SD 1.63) years with extremes ranging from 1 month to 62 years. The RF was positive for 222 patients (79.3%). The ACPA was positive for 178 patients (63.6%). The mean C-reactive protein (CRP) was 19.61 (SD 23.37) mg/L with extremes ranging from 0.2 to 189 mg/L.

**Table 1:** The main patients’ characteristics and disease features

Parameter		Numerical data (%)
**Sex distribution**	Female Male	233 (83.2) 47 (16.8)
**Age**	(years) ± SD	58.07 ± 11.12
**Age at diagnosis**	(years) ± SD	44.65 ± 13.78
**Disease duration**	(years) ± SD	14.74 ± 1.63
**DAS28 (CRP)**		4.76 ± 1.19
**HAQ**		1.4 ± 0.5
**Immunologic profile**	RF + ACPA +	222 (79.3) 178 (63.6)
**Extra-articular manifestations**	Pulmonary involvement Ocular involvement Pericarditis Kidney involvement Systemic vasculitis	136 (48.6) 119 (87.5) 22 (16.1) 6 (4.4) 5 (3.6) 3 (2.2)
**Coxitis**		34 (12.1)
**Atlanto-axial dislocation**		43 (15.4)

**DAS28**: disease activity score 28; **CRP**: C-reactive protein; **HAQ**: Health Assessment Questionnaire; **RF**: rheumatoid factor; **ACPA**: anti-citrullinated protein antibodies; **SD**: standard deviation

Regarding the disease activity, the mean DAS28 (CRP) was 4.76 (SD 1.19) with extremes varying from 2.05 to 7.47. The distribution of patients according to their disease activity was as follows: 11 (3.7%) in remission, 25 (9.0%) with low disease activity, 115 (41.4%) with moderate activity, and 129 (45.9%) with high disease activity. The mean HAQ was 1.4 (SD 0.5) with extremes ranging from 0.3 to 2.2. Among the RA cohort, 136 patients (63.0%) presented extra-articular manifestations: pulmonary involvement (n = 119, 42.5%) (Non-specific interstitial pneumonia (n = 46 (39.0%)), nodular lung disease (n = 29 (24.6%)), usual interstitial pneumonia (n = 21 (17.3%)), bronchiolitis obliterans organizing pneumonia (n = 9 (7.2%)), pleural nodules (n = 9 (7.2%))), rheumatoid nodules (n = 17, 6.1%), ocular involvement (n = 22, 7.9%), pericarditis (n = 6, 2.1%), kidney involvement (n = 5, 1.8%) (4 cases of glomerulonephritis and one case of amyloidosis), and systemic vasculitis (n = 3, 1.1%).

Pelvic radiographs showed coxitis in 34 patients (12.1%). Coxitis was unilateral in 32 patients and bilateral in 2 patients. During the disease course, 43 patients (15.4%) developed atlanto-axial dislocation. Regarding therapy, the use of corticosteroid was noted in 245 patients (87.7%). csDMARDS were prescribed as follows: Methotrexate (n = 246 (87.8%)), Sulfasalazine (n = 32 (11.5%)), Leflunomide (n = 5 (1.8%)), and Hydroxychloroquine (n = 8 (3.0%)). bDMARDs were prescribed for 72 patients (25.7%).

### Comorbidities among patients with RA

Comorbidities were noted in 133 patients (47.5%). Multimorbidity was identified at 60 patients (21.4%). The distribution of patients according to their different comorbidities is summarized in Table 2. The most frequent comorbidities were: hypertension (n = 84 (30.0%)), diabetes (n = 52 (18.6%)), lung disease (n = 18 (6.4%)) and cardio-vascular disease (n = 15 (5.3%)). The mean comorbidity score according to the RDCI was 1.05 (SD 1.23) with extremes ranging from 0 to 6.

**Table 2:** Distribution of patients according to their comorbid conditions

Comorbid condition		Number	Proportion (%)
**Lung disease**	Asthma COPD Bronchiectasis Others	18 5 3 5 5	6.4 1.7 1.1 1.7 1.7
**Cardio-vascular disease**	Myocardial infraction Stroke Other CV disease	15 5 8 2	5.3 1.7 2.8 0.7
**Hypertension**		84	30
**Diabetes**		52	18.5
**Fracture**		1	0.3
**Depression**		9	3.2
**Ulcer or stomach problem**		8	2.8
**Cancer**		3	1.1

RDCI was positively correlated with age (p < 0.001, r = 0.359). It was significantly increased in patients aged 65 years or over (2.1 (SD 1.4) [CI 95%:1.56–2.4] vs. 1.5 (SD 1.2), p = 0.009). RCDI has a tendency to be different in terms of gender (F: 1.5 (SD 1.2) vs. M: 1.8 (SD 1.3), p = 0.058). Regarding disease characteristics, RDCI was correlated with age at disease onset (p = 0.001, r = 0.266). It was higher in patients whose age of disease onset exceeds 40 years (1.8 (SD 1.3) [CI 95%: 1.36–1.88] vs. 1.5 (SD 1.2), p = 0.007). RDCI was not correlated to the disease duration (p = 0.263, r = 0.091). We did not find a difference in RDCI according to the immunological profile (seropositive: 1.6 (SD 1.2) vs. seronegative: 1.4 (SD 1.1), p = 0.968) and (immunopositive: 1.6 (SD 1.2) vs. immunonegative: 1.4 (SD 1.2), p = 0.887). RDCI was not correlated neither with the DAS28 (CRP) (p = 0.719, r = 0.035) nor with the HAQ (p = 0.444, r=-0.258).

The association between RDCI and main disease characteristics is summarized in Table 3. The comorbidity index was associated with the presence of extra-articular manifestations (p < 0.001); and significantly higher in patients presenting pulmonary involvement (p < 0.001) and ocular involvement (p = 0.002). RDCI was increased significantly but with a small difference in erosive RA (p = 0.006), in case of the presence of atlanto-axial dislocation (p = 0.014), and with coxitis (p = 0.029).

**Table 3:** Relation between RDCI and the main disease characteristics

Parameter	Mean RDCI (SD)	IC à 95%	p-value
**Age** ** < 40 years** ** 40–59 years** ** 60–79 years** ** > 80 years**	0.3 (SD 0.9) 0.8 (SD 1.0) 1.5 (SD 1.2) 2.4 (SD 2.6)	-0.11-0.77 0.70–1.08 1.27–1.74 -0.84-5.64	< 0.001
**Patient aged 65 years or over** ** Yes** ** No**	2.1 (SD 1.4) 1.5 (SD 1.2)	1.56–2.40 0.70–1.91	0.009
**Gender** ** Male** ** Female**	1.8 (SD 1.3) 1.5 (SD 1.2)	0.89–1.73 0.88–1.21	0.058
**Age at disease onset**	-	-	0.001
**Age at disease onset over 40 years** ** Yes** ** No**	1.8 (SD 1.3) 1.5 (SD 1.2)	1.36–1.88 0.67–1.37	0.007
**Disease duration**	-	-	0.263
**Seropositive RA** ** Yes** ** No**	1.6 (SD 1.2) 1.4 (SD 1.1)	0.91–1.26 0.75–1.41	0.968
**Immunopositive RA** ** Yes** ** No**	1.6 (SD 1.2) 1.4 (SD 1.2)	0.83–1.21 0.96–1.49	0.887
**DAS 28 (CRP)**	-	**-**	0.719
**HAQ**	-	**-**	0.444
**Extra-articular manifestations** ** Yes** ** No**	2.1 (SD 1.4) 0.5 (SD 0.7)	0.97–1.97 0.27–0.85	< 0.001
**Pulmonary involvement** ** Presence** ** Absence**	2.2 (SD 1.5) 0.4 (SD 0.6)	1.04–2.11 0.27–0.8	0.001
**Cardiac involvement** ** Presence** ** Absence**	2 (SD 1.5) 1.4 (SD 1.3)	0.37–3.63 1.14–1.66	0.297
**Ocular involvement** ** Presence** ** Absence**	2.7 (SD 1.6) 0.8 (SD 0.9)	1.12–2.77 0.46–1.03	0.002
**Kidney involvement** ** Presence** ** Absence**	1.3 (SD 1.1) 1.9 ± 1.3	-0.54-5.2 1.19–1.74	0.858
**Rheumatoid nodules** ** Presence** ** Absence**	2 (SD 1.4) 1.7 (SD 1.3)	-11.71-13.71 0.76–1.44	0.309
**Systemic vasculitis** ** Presence** ** Absence**	1.6 (SD 2) 1.8 (SD 1.3)	-23.41-27.41 0.78–1.45	0.317
**Radiographic erosions** ** Presence** ** Absence**	1.6 (SD 1.2) 0.7 (SD 0.8)	0.95–1.35 0.66–1.28	0.006
**Coxitis** ** Yes** ** No**	2.4 (SD 1.5) 1.4 (SD 1.1)	0.63–1.79 0.88–1.18	0.029
**Atlanto-axial dislocation** ** Yes** ** No**	1.9 (SD 1.3) 1.5 (SD 1.2)	0.70-2.00 0.58–1.37	0.014
**bDMARDs** **csDMARDs**	2.8 (SD 1.6) 1.0 (SD 1.0)	1.25–2.45 0.82–1.14	0.021

**RDCI**: Rheumatic Disease Comorbidity Index; **DAS28 (CRP)**: Disease activity scale (C-reactive protein); **HAQ**: Health assessment questionnaire; **bDMARDs**: biologic Disease-modifying antirheumatic drugs; **csDMARDs**: conventional synthetic Disease-modifying antirheumatic drugs **p**: statistical significance; **SD**: Standard deviation; **IC**: confidence interval

Regarding therapy regimen, RDCI was statistically higher in patients treated with bDMARDS versus patients receiving csDMARDS (p = 0.021).

On multivariable analysis, associated factors to the RDCI were: the presence of pulmonary involvement (p = 0.048, B = 0.975) and the presence of ocular involvement (p = 0.047, B = 1.033) (Table 4**)**.

**Table 4:** Data from multivariable analysis

Parameter	B	t	P-value	95% Confidence Interval	
				**Inferieur**	**Superieur**
Pulmonary involvment	0.975	2.054	0.048	**0.008**	**1.942**
Ocular involvment	1.033	2.064	0.047	**0.013**	**2.052**
Cardiac involvment	1.290	1.296	0.204	-0.738	3.319
Kidney involvment	1.004	0.724	0.474	-1.822	3.829
Presence of rheumatoid nodules	0.639	0.415	0.681	-2.493	3.770
Systemic vasculitis	-1.602	-0.759	0.454	-5.902	2.698
Radiographic erosions	0.888	1.609	0.117	-0.236	2.011
Atlanto-axial dislocation	-0.591	-1.170	0.251	-1.619	0.438
Presence of coxitis	0.137	0.257	0.799	-0.949	1.223

## Discussion

This study highlighted the association between comorbidities and different RA outcomes patients. About half of our patients have comorbidities (47.5%) with a high percentage of multimorbidity. Comorbidities may precede or appear during the course of the disease. Some studies noted that the burden of comorbidity is common before and after RA diagnosis, and the rate of accumulation accelerates after RA diagnosis [21, 22]. Our results were close to the reported scores of RDCI in RA [14, 21]. In this study, hypertension was the most common comorbidity as it has been reported in other studies [3, 15]. Other comorbidities such as diabetes, respiratory diseases, cardiovascular diseases, stroke, and depression are also frequent in RA [22].

Comorbidities may influence patients’ disability as well as frequency of hospitalization and mortality [9]. In fact, cardiovascular and respiratory diseases exposed RA patients to frequent hospitalizations and may be life threatening [10]. However, depression is commonly associated with disability [11]. The impact of comorbidities on RA outcomes is rarely studied using validated instruments such as the RDCI [13, 21].

In the present study we looked for the association between RDCI and RA characteristics.

In concordance with previous literature data, we found that RDCI was positively correlated with age and age at disease onset [17, 23, 24]. We demonstrated that RDCI was also higher in aged patients and in patients whose age of disease onset exceeds 40 years. The demographic profile of RA has changed with increased aged patients, making the prevalence of comorbidities also increased [25, 26].

Other factors related to the rheumatic disease may interfere and vary with comorbidities. Tidblad et al. found that the serological status may influence on patients’ comorbidities [24]. Seropositive profile was accompanied by high prevalence of pulmonary comorbidities such as chronic obstructive pulmonary disease (COPD). Smoking is a major factor of COPD. Smoking has an important link with RF [27]. However, in our study, data about smoking were not mentioned. It may be a limitation of our study. From our results, there was not a significant association between RDCI and the immunological profile of patients.

Moreover, extra-articular manifestations in RA have risen with an increased mortality rate in severe forms [28]. Their occurrence is due to several mechanisms. The role of immune system in their development is well established. This intervention is mediated by pro-inflammatory cytokines such as Tumor Necrosis Factor alpha, Interleukin 1, and Interleukin 6 [29].

In our study, comorbidity index was significantly higher in the presence of extra-articular manifestation such as pulmonary and ocular involvement. In fact, pulmonary involvement in RA and comorbidities may share common pathogenic mechanism. Some diseases, such as cardiovascular pathologies and diabetes that participate in ageing phenomenon of the body, are associated with telomeres shortening [30]. Telomeres are repeated sequences of nucleotides which protect chromosomes from degradation [31, 32]. Telomere shortening is also observed in RA due to the systemic inflammation and the effect of auto-antibodies [33]. So, we suggest that comorbidities and inflammation may worsen cellular ageing and accelerate the development of extra-articular manifestations. As in the study conducted by Nikiphorou et al., RCDI increased during the follow-up with a higher incidence of pulmonary involvement [22].

In addition to that, comorbidities have been reported to be associated with functional disability and deterioration of quality of life of RA patients [34, 35]. But, there is a paucity of data concerning the effect of comorbidities on irreversible part of disability assessed by structural damage. To our knowledge our study is the first to show a association between RDCI and joint destruction (erosive forms of RA, the presence of coxitis, and atlanto-axial dislocation). RDCI was higher in erosive RA, in case of coxitis, and atlanto-axial dislocation with a smaller difference. It means that a smaller increase may modify the disease prognosis. Elena Nikiphorou et al. used the need for joint surgery as a marker to evaluate joint destruction and concluded that to there was no association between RDCI and joint surgery [22].

As mentioned above in our results, comorbidities were not associated with the disease duration, the disease activity or with the functional impairment. This may be explained by the moderate average of the DAS28 and the HAQ. In the other hand, comorbidities were associated with the use of biologic drugs. In the literature, multimorbidity rise up the complexity of the disease management and it was shown to affect negatively therapeutic target within one year after initiating DMARDs [36]. Although, in the study conducted by Ellerby et al., investigating the effect of obesity and comorbidity on achieving remission, comorbidity scores were significantly higher in patients who have never achieved remission during 3 years of follow-up [37]. In a similar vein, Batko et al. have noted a doubled prevalence of comorbidities and a higher RDCI in patients with higher disease activity [38]. Also, an elevated RDCI score has been proven to be a predictor of biologics’ discontinuation and a negative predictor of achieving moderate to good response [14]. This positive association in our results may be explained by the severity of RA in patients receiving bDMARDs.

The main limitation of the present study is the selection bias since we included hospitalized RA patients with more severe disease. Thus, these results may not be generalizable to other populations. Other greater studies are needed to approve our findings.

## Conclusion

In this study, we elaborated important findings expanding our current knowledge and enriched the available literature data. It seems clear that elderly patients face more comorbidities. Based on our results, we confirmed the association of comorbidities with severe outcomes and signs of poor prognosis such as structural damages for patients with RA. With increasing life-expectancy and improved control of joint disease in RA, the relative importance of comorbidity in RA is increasing and can be a threat to the improvement in the long-term prognosis in RA patients. Thus, we highlight the importance of managing comorbidities in this population as part of managing RA.

## Data Availability

The data and supportive information is available within the article.

## Abbreviations

ACPA: anti-citrullinated protein antibodies RA:Rheumatoid arthritis
bDMARD: biologic Disease-modifying antirheumatic drug
CCI: Charlson Comorbidity Index
CI: confidence interval
csDMARD: conventional synthetic Disease-modifying antirheumatic drug
CRP: C-reactive protein
DAS28: disease activity score 28
ECI: Elixhauser Comorbidity Index
HAQ: Health Assessment Questionnaire
RDCI: Rheumatic Disease Comorbidity Index
RF: rheumatoid factor.
COPD: chronic obstructive disease
